# Predictors for Depression, Sleep Disturbance, and Subjective Pain among Inpatients with Depressive Disorders during the COVID-19 Pandemic: A Cross-Sectional Study

**DOI:** 10.3390/ijerph18126523

**Published:** 2021-06-17

**Authors:** Dian-Jeng Li, Su-Ting Hsu, Frank Huang-Chih Chou, Li-Shiu Chou, Kuan-Ying Hsieh, Wei-Tsung Kao, Guei-Ging Lin, Wei-Jen Chen, Che-Hun Liao, Joh-Jong Huang

**Affiliations:** 1 Department of Addiction Science, Kaohsiung Municipal Kai-Syuan Psychiatric Hospital, Kaohsiung 80276, Taiwan; u108800004@kmu.edu.tw (D.-J.L.); 030854@gmail.com (W.-T.K.); 2 Department of Nursing, Meiho University, Pingtung 91200, Taiwan; 3 Department of Community Psychiatry, Kaohsiung Municipal Kai-Syuan Psychiatric Hospital, Kaohsiung 80276, Taiwan; hsu.suting@gmail.com; 4 Department of Family Medicine, Kaohsiung Medical University Hospital, Kaohsiung 80708, Taiwan; 5 Office of Dean, Kaohsiung Municipal Kai-Syuan Psychiatric Hospital, Kaohsiung 80276, Taiwan; 6 Office of Vice Dean, Kaohsiung Municipal Kai-Syuan Psychiatric Hospital, Kaohsiung 80276, Taiwan; lishiu.chou@msa.hinet.net; 7 Department of Child and Adolescent Psychiatry, Kaohsiung Municipal Kai-Syuan Psychiatric Hospital, Kaohsiung 80276, Taiwan; isanrra@gmail.com; 8 Department of Sports, Health and Leisure and Graduate Institute of Sports, Health and Leisure, Cheng Shiu University, Kaohsiung 83347, Taiwan; 9 Department of Nursing, Kaohsiung Municipal Kai-Syuan Psychiatric Hospital, Kaohsiung 80276, Taiwan; t1921118@yahoo.com.tw; 10 Department of Neuropsychiatry, Kaohsiung Municipal Kai-Syuan Psychiatric Hospital, Kaohsiung 80276, Taiwan; chenweijenmail@gmail.com; 11 Graduate Institute of Counseling Psychology and Rehabilitation Counseling, National Kaohsiung Normal University, Kaohsiung 82444, Taiwan; 12 Department of Business Management, National Sun Yat-sen University, Kaohsiung 80424, Taiwan; smartmean@yahoo.com.tw; 13 Department of Health, Kaohsiung City Government, Kaohsiung 80251, Taiwan

**Keywords:** depression, sleep disturbance, pain, social impact, psychiatric inpatient

## Abstract

The coronavirus disease 2019 (COVID-19) pandemic can have a negative impact on patients with mood disorders. The aim of this study is to explore the societal influence of COVID-19 and associated impacts on levels of depression, sleep disturbance, and subjective pain among patients with mood disorders. This cross-sectional study recruited inpatients with depression and bipolar disorder. Levels of depression, sleep disturbance, subjective pain, and related demographic variables were collected through self-reported questionnaires. Potential factors associated with levels of depression, sleep disturbance, and subjective pain were identified using univariate linear regression and further entered into a stepwise multivariate linear regression model to identify the independent predictors. A total of 119 participants were included in the analysis, of whom 50.42% had bipolar disorder and 49.58% had unipolar depression. Multivariate analysis showed that a higher level of depression was associated with female subjects, subjects with partners, present history of psychological trauma, and drinking alcohol. Sleep disturbance was associated with subjects with partners and drinking alcohol. A higher level of subjective pain was associated with a higher level of social anxiety and a history of psychological trauma. The current study identified several predictors of psychological burden and subjective pain among inpatients with depression during the COVID-19 pandemic. Further investigations are warranted to extend the application and generalizability of our results.

## 1. Introduction

### 1.1. Psychological Impact of COVID-19

Coronavirus disease 2019 (COVID-19) has spread worldwide since the end of 2019 and has deeply affected people’s daily lives, including health burden and social-economic wellbeing [1]. With uncertainty of the disease and a massive amount of information published in the media, COVID-19 has also resulted in general alarm in the public [2]. An online study revealed that COVID-19 contributed to anxiety, depressive symptoms, and sleep disturbance in the general public [3], and another study reported that up to 25% of college students suffer from symptoms of anxiety [4]. Moreover, a meta-analysis, involving a total of 54,231 participants from 13 nations, reported that the global pooled prevalence rate of sleep problems among all populations was 35.7% during the COVID-19 pandemic [5]. Several studies based on the general public in Taiwan have also demonstrated undesirable psychological impacts during the COVID-19 pandemic. Li et al. found associations between sleep disturbance and suicidal thoughts with multiple COVID-19-related factors, such as the impact of COVID-19 on social interaction, more academic/occupational interference due to COVID-19, and lower COVID-19-specific support [6]. Another study indicated that a higher level of subjective mental health was associated with confidence about COVID-19 [7]. Therefore, the multi-dimensional impacts of COVID-19 on psychological health are an important issue. 

### 1.2. Massive Impact of COVID-19 on Patients with Mental Disorders

More attention should be paid to patients with mental disorders during the COVID-19 epidemic. A previous study reported that patients with mental health disorders suffer from an increased risk of infectious diseases [8], and discrimination associated with mental illness may make it difficult for patients to receive timely treatment for COVID-19. Furthermore, psychological stress due to COVID-19 could have a major influence on the progress of mental disorders because of poorer tolerability to stress compared with the general population [9].

Few studies have discussed these psychological impacts on patients with mental disorders. A previous study recruiting patients with schizophrenia reported that those who were suspected of having COVID-19 in the isolation ward had significantly higher levels of distress, depression, and poorer sleep quality compared to patients in uninfected groups [10]. Another pilot study reported that 38% (12 out of 32) of patients with eating disorders reported a worsening of symptoms and that 56.2% suffered from additional anxiety symptoms [11]. Compared to healthy control groups, patients with depressive and bipolar disorders have been shown to have heightened psychological distress and adverse changes to lifestyle behaviors [12]. In addition, subjective pain may also play an important role in the psychological impact of the COVID-19 pandemic. Subjective pain has been associated with mental disorders, and a higher level of psychological pain has been significantly associated with affective disorder, anxiety disorder [13], and even suicidality [14]. Psychological stress can affect sensitivity to thermal stimuli and enhance pain experience [15]. Moreover, acute stress can also exacerbate the intensity of chronic pain [16]. Given the enormous impact on psychological stress caused by COVID-19, measuring subjective pain may help to estimate the psychological effect among patients with mental disorders during the COVID-19 epidemic.

### 1.3. Aim of the Current Study

The psychological impacts of COVID-19 among patients with mental disorders have been preliminarily identified. However, the impacts of COVID-19 on social interaction and infectious diseases have not been explored in patients with mental disorders. A previous study reported that mental health problems were associated with social interaction and the ability to cope with COVID-19 among the general public in Taiwan [6]. Since a number of studies have explored the undesirable effects of COVID-19 on the public, further investigations on the psychological impacts on patients with mental disorders are needed. Researchers have compared differences between patients with mood disorders and healthy controls with regard to the impact of COVID-19 [12]. However, studies regarding associations between mood symptoms or subjective pain and social impacts of COVID-19 are lacking. Therefore, we conducted this cross-sectional study to comprehensively investigate the associations between the severity of mood symptoms and the social impacts of COVID-19. The aim of this study was to explore whether the levels of depression, sleep disturbance, and subjective pain among patients with depressive disorder were associated with multiple factors, including the social impact of the COVID-19 pandemic.

## 2. Methods

### 2.1. Participants and Ethics

The current study was conducted in Kaohsiung Municipal Kai-Syuan Psychiatric Hospital (KSPH). Participants were recruited through printed advertisements posted in the public area in the hospital. The recruitment period was from 9 May 2020, to 31 May 2020. Patients who intended to participate in this survey could inform their medical staff, and then research assistants would contact them. This study is a cross-sectional survey with paper-and-pencil questionnaires, and research assistants individually explained the procedures to the participants in order to complete the research questionnaires. The inclusion criteria were: (1) participants who were diagnosed with major depressive disorder or bipolar depression with DSM-5 according to the medical records, (2) admitted to the acute or chronic ward of KSPH, (3) could understand the objective of the study and follow the instructions from research assistants, (4) were aged between 20 to 80 years, and (5) signed informed consent before filling in the questionnaire. Data with missing values or from those who could not complete the questionnaire were excluded. In addition, patients with severe symptoms of depression or predominant decline of cognitive function that might interfere with the studying process were not enrolled. The sample size was determined using G-Power software with the statistical method of linear multiple regression [17]. The alpha value was set at 0.05, with a power of 0.95 and effect size of 0.6 with a medium effect [18]. The minimum required sample size was 85 patients in total. Considering the possibility of dropout, the required sample size was preliminarily set as 130 subjects. This study was approved by the Institutional Review Board of KSPH (KSPH-2020-03) and conducted according to the current revision of national legal requirements (Human Subjects Research Act, Taiwan).

### 2.2. Measure

#### 2.2.1. Depression Scales from the Disaster-Related Psychological Screening Test

The level of depression was measured using three questions from the Disaster-Related Psychological Screening Test (DRPST). The DRPST has been shown to be reliable and well-validated to rapidly screen for major depressive disorder or post-traumatic stress disorder (PTSD) after a disaster [19,20]. Three items were used to estimate the status of depressed mood, fatigue or loss of energy, and worthlessness which had persisted for more than 2 weeks in the last 1 month. Each item was rated on a 2-point Likert scale, with scores ranging from 0 (no) to 1 (yes). Higher total scores of the three items indicated higher levels of depression. Details of the questionnaire are listed in Supplementary Table S1. 

#### 2.2.2. Sleep Disturbance Scales from the Pittsburgh Sleep Quality Index

The Pittsburgh Sleep Quality Index (PSQI) was initially developed to measure sleep quality in clinical populations, and it has been shown to have good validity and reliability [21]. Four items selected from the PSQI were used to estimate the level of sleep disturbance: difficulty falling asleep, waking up in the middle of the night, subjective sleep quality, and enthusiasm in the recent 1 month (Supplementary Table S1). Each item was rated on a 4-point Likert scale, with scores ranging from 1 to 4. Higher total scores of the four items indicated more severe sleep disturbance.

#### 2.2.3. Subjective Pain Measured Using the 12-Item Short Form Survey Version 2

The 12-item Short Form Survey version 2 (SF-12v2) is based on scoring coefficients derived from version 1 of the SF-36. It was developed to rapidly estimate general health status and has since been well validated [22]. One item was selected from the SF-12v2 to measure the level of interference caused by subjective pain in the past 1 month (Supplementary Table S1). This item was graded on a 5-point Likert scale, with scores ranging from 1 (not at all) to 5 (extremely). A higher score represented a higher level of interference.

#### 2.2.4. Subscales from the Societal Influences Survey Questionnaire 

The Societal Influences Survey Questionnaire (SISQ) was constructed to measure the psychological and social impact on individuals during the COVID-19 pandemic. With acceptable validity and reliability, the 15-item SISQ contains five categories of assessment: social distance, social anxiety, social desirability, social information, and social adaptation [23]. Ten of the questions were selected in the current study with four domains: social distance, social anxiety, social information, and social adaptation (Supplementary Table S1). Each question was scored on a 4-point Likert scale, with scores ranging from 1 (never) to 4 (often). Higher total scores of each domain (social distance, social anxiety, social information, and social adaptation) indicated higher compliance to maintain social distance, higher level of anxiety due to COVID-19, more desire to seek related information, and more awareness of progress of the pandemic overseas, respectively.

#### 2.2.5. Demographic Characteristics

Data were recorded as continuous variables for the participants’ age and educational level (years). Categorical variables included sex, occupational status, marital status, religion, history of psychological trauma, smoking (yes or no), drinking alcohol (≥3 times per week or not), exercise (≥3 days per week or not), regular diet (three or four meals a day, ≥5 days per week or not), and history of chronic medical diseases. 

### 2.3. Statistical Analysis

Descriptive analysis was performed on the demographic variables. Marital status was transformed into a dichotomous variable as “with partner” (married and cohabiting) or “without partner” (single, widowed, and divorced). Histories of chronic medical diseases and psychological trauma were also transformed into dichotomous variables as “yes” or “no”. Since the questionnaires in the current study were all validated, Cronbach’s α was used to test the reliability of each scale containing more than two questions. Univariate and stepwise multiple linear regression analyses were conducted to ascertain whether the independent factors were associated with dependent variables, including level of depression, sleep disturbance, and subjective pain. The alpha level was set at 0.05. 

The normality of dependent variables was checked using the Kolmogorov–Smirnov test. Because non-normally distributed samples were identified with the significance of the test (*p* < 0.001), bootstrapping multiple linear regression with 1000 bootstrap samples was used to verify the results from the stepwise multivariate linear regression analysis. In the bootstrapping method, 95% confidence intervals were used to determine statistical significance, as this could qualify the stability of the regression coefficients and reduce the length of the confidence interval [24]. When the 95% confidence interval of a regression coefficient did not contain zero, the variable was statistically significant. In addition, the number of bootstrap samples was set as 1000 to obtain a sufficiently accurate 95% bootstrap percentile [25]. All data were processed using SPSS version 23.0 for Windows (SPSS Inc., Chicago, IL, USA).

## 3. Results

### 3.1. Summary of Demographic Analysis and Reliability of the Questionnaires

A total of 122 subjects completed the survey. We excluded three subjects who did not complete all questions, and the remaining 119 subjects (58 females and 61 males; mean age 46.67 ± 12.39 years) were entered into the analysis. Sixty (50.42%) of the participants reported that they had been diagnosed with bipolar disorder, and 59 (49.58%) reported they had been diagnosed with unipolar depressive disorder. Among the dependent variables, the mean scores of depressions, sleep disturbance and subjective pain were 1.76 ± 1.22, 9.82 ± 3.49, and 2.37 ± 1.24, respectively. The reliability estimated with Cronbach’s α for scales of depression, sleep disturbance, social distance, social anxiety, social information, and social adaptation were 0.77, 0.83, 0.81, 0.61, 0.65, and 0.68, respectively. All of the values were above 0.6, indicating an acceptable range [26]. The remaining summaries of characteristics are listed in Table 1. The distribution of marital status, history of psychological trauma, and chronic medical diseases are presented in Supplementary Tables S2–S4.

### 3.2. Predictors of the Level of Depression

Table 1 shows the results of univariate and stepwise multivariate linear regression analyses. The results of univariate linear regression analysis revealed that a higher level of depression was significantly associated with a higher level of social anxiety (standardized coefficient β = 0.23; *p* = 0.012), female subjects (β = 0.24; *p* = 0.009), subjects with partners (β = 0.18; *p* = 0.0497), history of psychological trauma (β = 0.25; *p* = 0.006), and drinking alcohol (β = 0.18; *p* = 0.049).

The stepwise linear regression analysis excluded the level of social anxiety. The results of the final model were as follows: female subjects (β = 0.22; *p* = 0.013), subjects with partners (β = 0.18; *p* = 0.035), history of psychological trauma (β = 0.24; *p* = 0.005), and drinking alcohol (β = 0.20; *p* = 0.018). To verify whether these results were influenced by the non-normality of outcome variables, multivariate linear regression with 1000 bootstrapping samples was conducted. The results of bootstrapping multiple linear regression demonstrated the same patterns of independent associators for the level of depression (Supplementary Table S5). 

### 3.3. Predictors for the Level of Sleep Disturbance

The results of univariate and stepwise multivariate linear regression analyses are presented in Table 2. The results of univariate linear regression analysis demonstrated that a higher level of sleep disturbance was significantly associated with a higher level of social anxiety (β = 0.23; *p* = 0.012), higher level of social information (β = 0.27; *p* = 0.007), higher level of social adaptation (β = 0.28; *p* = 0.006), female subjects (β = 0.24; *p* = 0.020), subjects with partners (β = 0.22; *p* = 0.034), and drinking alcohol (β = 0.25; *p* = 0.015).

The stepwise linear regression analysis excluded the level of social information, social adaptation, and female subjects. The results of the final model of the significant factors were as follows: higher level of social anxiety (β = 0.33; *p* = 0.001), subjects with partners (β = 0.23; *p* = 0.015), and drinking alcohol (β = 0.26; *p* = 0.006). Multivariate linear regression with 1000 bootstrapping samples was conducted to verify the non-normality of the samples. However, the level of social anxiety was excluded from bootstrapping analysis. The remaining factors examined by bootstrapping regression were subjects with partners and drinking (Supplementary Table S6).

### 3.4. Predictors for the Level of Subjective Pain

The results of univariate linear regression demonstrated that a higher level of subjective pain was significantly associated with a higher level of social anxiety (β = 0.38; *p* < 0.001), higher level of social information (β = 0.25; *p* = 0.007), higher level of social adaptation (β = 0.22; *p* = 0.015), history of psychological trauma (β = 0.27; *p* = 0.033), and presence of a chronic medical disease (β = 0.24; *p* = 0.009) (Table 3).

The level of social information, social adaptation, and history of chronic medical diseases were excluded from stepwise linear regression analysis. The remaining factors were a higher level of social anxiety (β = 0.34; *p* < 0.001) and history of psychological trauma (β = 0.19; *p* = 0.028). After verification with 1000 bootstrapping multiple linear regression, the significant factors were the same as in stepwise linear regression (Supplementary Table S7).

## 4. Discussion

### 4.1. Main Findings of the Current Study

To the best of our knowledge, this is the first study to comprehensively assess the impacts of social influence and related factors on the level of depression, sleep disturbance, and subjective pain in patients with depressive disorders. After verification with stepwise regression analysis, a higher level of depression was associated with female subjects, subjects with partners, history of psychological trauma, and drinking alcohol. Sleep disturbance was associated with subjects with partners, drinking alcohol, and a higher level of social anxiety (not verified with bootstrap regression). A higher level of subjective pain was associated with a higher level of social anxiety and history of psychological trauma. Although social anxiety was not entirely verified with both stepwise and bootstrap linear regression analyses, social anxiety had a greater impact on a higher level of sleep disturbance and physical pain than the other categories of the SISQ. 

### 4.2. Factors Associated with Level of Depression and Sleep Disturbance

Faced with uncertainty and threat due to COVID-19, people may suffer from mental health problems such as anxiety [23]. Another Taiwanese study reported that excessive anxiety was associated with lower psychological wellbeing during the COVID-19 pandemic [27]. The current study further extends this implication to patients with depressive or bipolar disorder, indicating the significant association between concerns over COVID-19 and depressive and sleep problems. Patients with depressive or anxiety disorder have been reported to demonstrate more serious concerns about their physical health, impulsivity, sleep disturbance, and intense suicidal ideation than healthy controls [28]. Therefore, assessing anxiety levels due to COVID-19 is crucial to identify undesirable impacts on depressive symptoms and sleep problems at an early stage in patients with mood disorders. 

In this study, social information and social adaptation were potentially associated with sleep disturbance, even though both have been shown to be protective factors to actively cope with COVID-19 by obtaining knowledge and awareness of the progress overseas [23]. Nevertheless, such behaviors may be associated with psychological burden. For instance, excessive media exposure to COVID-related news has been shown to trigger anxiety and stress responses among the general public [29]. In addition, to enhance active coping with COVID-19, psychological interventions are also crucial for patients with mood disorders.

Our results showed that the female subjects were significantly associated with a higher level of depression and potentially with sleep disturbance. Females have been reported to be at risk of sleep problems and anxiety during the COVID-19 pandemic [30]. In addition, females have been reported to have higher levels of depressive symptoms than males among patients with major depressive disorders [31]. Compared to males, females are socialized to express their emotions more strongly and to have negative views of their health, resulting in psychological problems [32]. Hence, the gender difference in depression or sleep problems is supported by previous evidence. In addition, drinking alcohol more than three times per week was associated with sleep disturbance and higher level of depression. A previous study identified a link between alcohol use and depression [33], and the sleep cycle has also been reported to be disturbed by alcohol intake [34]. Our findings highlight the undesirable impact of alcohol on patients with mood disorders and support the potential risk of increased alcohol use after a disaster [35]. Furthermore, a history of psychological trauma was associated with a higher level of depression. Traumatic events are predominantly associated with later depression [36], and thus PTSD may emerge in the remission stage of COVID-19 for those facing major trauma, such as the loss of family members or close friends due to COVID-19. 

Finally, we found that married patients had higher levels of depression and sleep disturbance, in contrast to reports that being married is protective against depression [37]. It is possible that the married patients may have been worried about their partners due to COVID-19, thereby exacerbating symptoms of depression and sleep problems. Further studies are warranted to validate and explore the possible mechanisms.

### 4.3. Factors Associated with Subjective Pain

The level of subjective pain was positively correlated to the level of social anxiety in our patients. Subjective pain is associated with psychological distress [15]. Furthermore, depression, anxiety, and pain have high comorbidity and share similar neurophysiological mechanisms, such as dysregulation of the anterior cingulate cortex, insular cortex, and ventral tegmental area [38]. Glutamate signaling, especially signaling through AMPA receptors, has been shown to play a crucial role in regulating pain and emotions [39,40]. In addition, the potential effects of social information and social adaptation are thought to be mediated by excessive worry.

A history of psychological trauma also predicted a higher level of subjective pain in our patients. A previous study reported that gay and bisexual men who experienced homophobic bullying in childhood experienced more physical pain in adulthood than those who had not been bullied [41]. Another study focusing on patients with lower back pain indicated that a history of more traumatic events was significantly related to greater pain severity, pain interference, and depressive symptoms [42]. These findings are comparable to those of the current study. 

### 4.4. Limitations

The current study has several limitations that need to be addressed. First, the patients’ diagnoses were identified through self-reported questionnaires. Although they were inpatients hospitalized in a psychiatric hospital, it would have been better if they had been verified by research psychiatrists through a diagnostic interview. Second, the baseline severity of mood disorder was not assessed, which may have confounded the scoring of depression or sleep disturbance. However, we recruited those who could understand the objective of this study and follow instructions from the assistants. In addition, patients with severe symptoms of depression might not have enough ability (e.g., poor concentration) to complete the questionnaires, and they would have been excluded from the initial screen. Third, the cross-sectional design of this study limited causal inference for further interpretation. Fourth, the details of family members were not collected, and it may confound the association between marital status and sleep disturbance or depression. Fifth, details of religion (Buddhist, Christian, etc.) were not recorded in the questionnaires. Finally, a single-center study and a relatively small sample size of the current study may limit the generalizability and applicability to other populations.

## 5. Conclusions

This study identified several predictors for the levels of depression, sleep disturbance, and subjective pain among patients with depressive disorders during the COVID-19 pandemic. Our results showed associations between social impact and psychological burden. Therefore, the early identification of excessive worry about the pandemic and timely interventions are important for patients with depressive disorder. In addition, patients with a history of alcohol use and psychological trauma should also be carefully followed during the COVID-19 pandemic. Regular screening and prompt treatment for PTSD and related psychological burdens will be beneficial for patients with mental disorders. 

We propose several suggestions for further research, which could help extend the findings of the present study. Based on the significant predictors of sleep disturbance, depression, and subjective pain, the current study provided clues to the further study for more complex interaction between stressors and their predictors. For instance, the development of a mediation or moderated mediation model may be beneficial to understand the detailed etiology of the formation and interaction between factors. A study with longitudinal follow-up may be helpful to better understand the psychological and social impacts on patients at different stages of the pandemic. Moreover, additional assessments of PTSD and substance use can further explore the impacts of the COVID-19 pandemic. A more nuanced theoretical study may also be helpful to clarify the etiology of depression, sleep disturbance, and subjective pain within patients with depressive disorder during the COVID-19 pandemic.

## Figures and Tables

**Table 1:** Predictors for the level of depression examined by univariate linear regression and stepwise multivariate linear regression (N = 119).

Predictors			Univariate Regression				Stepwise Multivariate Regression			
	Mean	SD	β	t	95% CI	*p*	β	t	95% CI	*p*
Age (years)	46.67	12.39	0.06	0.69	−0.01, 0.02	0.493	-	-	-	-
Education level (years)	12.39	3.28	−0.17	−1.82	−0.13, 0.01	0.071	-	-	-	-
Social distance	10.52	3.89	0.05	0.54	−0.04, 0.07	0.588	-	-	-	-
Social anxiety	4.48	2.01	0.23	2.57	0.03, 0.25	**0.012**	0.15	1.66	-	0.10 ^a^
Social information	5.03	1.98	−0.01	−0.15	−0.12, 0.10	0.879	-	-	-	-
Social adaptation	5.07	2.28	0.02	0.26	−0.09, 0.11	0.794	-	-	-	-
	*n*	%	β	t	95% CI	*p*	β	t	95% CI	*p*
Sex										
Male	61	51.3	Ref	-	-	-	Ref	-	-	-
Female	58	48.7	0.24	2.64	0.14, 1.01	**0.009**	0.22	2.51	0.11, −0.93	**0.013**
Occupational status										
Unemployment	92	77.3	Ref	-	-	-	Ref	-	-	-
Employment	27	22.7	0.03	0.28	−0.46, 0.61	0.778	-	-	-	-
Marital status										
Without partner	103	86.6	Ref	-	-	-	Ref	-	-	-
With partner	16	13.4	0.18	1.98	0.001, 1.28	**0.0497**	0.18	2.13	0.05, 1.25	**0.035**
Religion										
Not religious	24	20.2								
Religious	95	79.8	0.12	1.34	−0.18, 0.92	0.182	-	-	-	-
Psychological trauma										
No	32	26.9								
Yes	87	73.1	0.25	2.82	0.21, 1.18	**0.006**	0.24	2.85	0.20, 1.13	**0.005**
Smoking										
No	86	72.3	Ref	-	-	-	-	-	-	-
Yes	33	27.7	0.03	0.34	−0.41, 0.58	0.734	-	-	-	-
Drinking (≥3 times per week)										
No	102	85.7	Ref	-	-	-	Ref	-	-	-
Yes	17	14.3	0.18	1.99	0.002, 1.25	**0.049**	0.20	2.39	0.12, 1.29	**0.018**
Exercise (≥3 days per week)										
No	38	31.9	Ref	-	-	-	-	-	-	-
Yes	81	68.1	0.07	0.76	−0.29, 0.66	0.448	-	-	-	-
Regular diets (≥5 days per week)										
No	22	18.5	Ref	-	-	-	-	-	-	-
Yes	97	81.5	−0.02	−0.26	−0.65, 0.50	0.794	-	-	-	-
Chronic disease (medical)										
No	46	38.7	Ref	-	-	-	-	-	-	-
Yes	73	61.3	0.14	1.52	−0.11, 0.80	0.132	-	-	-	-

β: standardized coefficients; t: T score; CI: Confidence interval; SD: Standard deviation; **Bolds:** *p* < 0.005; ^a^: excluded from stepwise regression.

**Table 2:** Predictors for level of sleep disturbance examined by univariate linear regression and stepwise multivariate linear regression (N = 119).

Predictors			Univariate Regression				Stepwise Multivariate Regression			
	Mean	SD	β	t	95% CI	*p*	β	t	95% CI	*p*
Age (years)	46.67	12.39	0.01	0.13	−0.06, 0.06	0.898	-	-	-	-
Education level (years)	12.39	3.28	−0.07	−0.70	−0.29, 0.14	0.483	-	-	-	-
Social distance	10.52	3.89	0.19	1.88	−0.001, 0.36	0.063	-	-	-	-
Social anxiety	4.48	2.01	0.32	3.29	0.22, 0.89	**0.001**	0.33	3.58	0.25, 0.88	**0.001**
Social information	5.03	1.98	0.27	2.73	0.13, 0.85	**0.007**	0.06	0.50	-	0.616 ^a^
Social adaptation	5.07	2.28	0.28	2.81	0.13, 0.74	**0.006**	0.14	1.14	-	0.257 ^a^
	*n*	%	β	t	95% CI	*p*	β	t	95% CI	*p*
Sex										
Male	61	51.3	Ref	-	-	-	Ref	-	-	-
Female	58	48.7	0.24	2.36	0.26, 3.05	**0.020**	0.18	1.91	-	0.059 ^a^
Occupational status										
Unemployment	92	77.3	Ref	-	-	-	-	-	-	-
Employment	27	22.7	0.09	0.83	−0.99, 2.34	0.409	-	-	-	-
Marital status										
Without partner	103	86.6	Ref	-	-	-	Ref	-	-	-
With partner	16	13.4	0.22	2.15	0.17, 4.11	**0.034**	0.23	2.49	0.46, 4.06	**0.015**
Religion										
Not religious	24	20.2	Ref	-	-	-	-	-	-	-
Religious	95	79.8	0.16	1.60	−0.34, 3.19	0.113	-	-	-	-
Psychological trauma										
No	32	26.9	Ref	-	-	-	-	-	-	-
Yes	87	73.1	0.18	1.76	−0.19, 3.15	0.081	-	-	-	-
Smoking										
No	86	72.3	Ref	-	-	-	-	-	-	-
Yes	33	27.7	0.40	0.39	−1.26, 1.87	0.701	-	-	-	-
Drinking (≥3 times per week)										
No	102	85.7	Ref	-	-	-	Ref	-	-	-
Yes	17	14.3	0.25	2.49	0.47, 4.17	**0.015**	0.26	2.83	0.73, 4.14	**0.006**
Exercise (≥3 days per week)										
No	38	31.9	Ref	-	-	-	-	-	-	-
Yes	81	68.1	−0.13	−1.23	−2.45, 0.58	0.223	-	-	-	-
Regular diets (≥5 days per week)										
No	22	18.5	Ref	-	-	-	-	-	-	-
Yes	97	81.5	−0.12	−1.17	−3.02, 0.78	0.246	-	-	-	-
Chronic disease (medical)										
No	46	38.7	Ref	-	-	-	-	-	-	-
Yes	73	61.3	0.14	1.31	−0.50, 2.43	0.194	-	-	-	-

β: standardized coefficients; t: T score; CI: Confidence interval; SD: Standard deviation; **Bolds:** *p* < 0.005; ^a^: excluded from stepwise regression.

**Table 3:** Predictors for level of physical pain examined by univariate linear regression and stepwise multivariate linear regression (N = 119).

Predictors			Univariate regression				Stepwise Multivariate Regression			
	Mean	SD	β	t	95% CI	*p*	β	t	95% CI	*p*
Age (years)	46.67	12.39	0.07	0.83	−0.01, 0.03	0.411	-	-	-	-
Education level (years)	12.39	3.28	−0.03	−0.33	−0.08, 0.06	0.742	-	-	-	-
Social distance	10.52	3.89	0.05	0.17	−0.01, 0.11	0.072	-	-	-	-
Social anxiety	4.48	2.01	0.38	4.49	0.13, 0.34	**<0.001**	0.34	3.90	0.10, 0.31	**<0.001**
Social information	5.03	1.98	0.25	2.76	0.04, 0.27	**0.007**	0.06	0.61	-	0.542 ^a^
Social adaptation	5.07	2.28	0.22	2.46	0.02, 0.22	**0.015**	−0.05	−0.44	-	0.664 ^a^
	*n*	%	β	t	95% CI	*p*	β	t	95% CI	*p*
Sex										
Female	61	51.3	Ref	-	-	-	-	-	-	-
Male	58	48.7	0.13	1.42	−0.13, 0.77	0.159	-	-	-	-
Occupational status										
Unemployment	92	77.3	Ref	-	-	-	-	-	-	-
Employment	27	22.7	0.11	1.24	−0.20, 0.87	0.217	-	-	-	-
Marital status										
Without partner	103	86.6	Ref	-	-	-	-	-	-	-
With partner	16	13.4	0.04	0.45	−0.51, 0.81	0.654	-	-	-	-
Religion										
Not religious	24	20.2	Ref							
Religious	95	79.8	0.13	1.46	−0.15, 0.97	0.148	-	-	-	-
Psychological trauma										
No	32	26.9	Ref				Ref	-	-	-
Yes	87	73.1	0.27	30.8	0.27, 1.25	**0.003**	0.19	2.23	0.06, 1.02	**0.028**
Smoking										
No	86	72.3	Ref	-	-	-	-	-	-	-
Yes	33	27.7	0.03	0.30	−0.43, 0.58	0.768	-	-	-	-
Drinking (≥3 times per week)										
No	102	85.7	Ref	-	-	-	-	-	-	-
Yes	17	14.3	0.09	0.99	−0.32, 0.97	0.322	-	-	-	-
Exercise (≥3 days per week)										
No	38	31.9	Ref	-	-	-	-	-	-	-
Yes	81	68.1	−0.03	−0.31	−0.56, 0.41	0.759	-	-	-	-
Regular diets (≥5 days per week)										
No	22	18.5	Ref	-	-	-	-	-	-	-
Yes	97	81.5	0.07	0.79	−0.35, 0.81	0.434	-	-	-	-
Chronic disease (medical)										
No	46	38.7	Ref	-	-	-	Ref	-	-	-
Yes	73	61.3	0.24	2.65	0.15, 1.05	**0.009**	0.16	1.86	-	0.066 ^a^

β: standardized coefficients; t: T score; CI: Confidence interval; SD: Standard deviation; **Bolds:** *p* < 0.005; ^a^: excluded from stepwise regression.

## Data Availability

The data presented in this study are available on request from the corresponding author.

## Supplementary Materials

The following are available online at https://www.mdpi.com/article/10.3390/ijerph18126523/s1, Table S1: Measures used in this study; Table S2: Distribution of marital status; Table S3: Distribution of events for psychological trauma; Table S4: Distribution of chronic disease (medical); Table S5: Predictors for the level of depression verified with multivariate linear regression with 1000 bootstrapping samples; Table S6: Predictors for the level of sleep disturbance verified with multivariate linear regression with 1000 bootstrapping samples; Table S7: Predictors for level of physical pain verified with multivariate linear regression with 1000 bootstrapping samples.
