# Supplementary material for: Predictors for Depression, Sleep Disturbance, and Subjective Pain among Inpatients with Depressive Disorders during the COVID-19 Pandemic: A Cross-Sectional Study
Source: Int J Environ Res Public Health. 2021 Jun 17;18(12):6523. doi: 10.3390/ijerph18126523 (PMC8296448; doi:10.3390/ijerph18126523)
Supplement: Supplementary file 1 [file ijerph-18-06523-s001.zip › ijerph-1253701-supplementary.pdf]

**Supplementary Table S1 Measures used in this study.**

| Measures | Items | Response scale |
|---|---|---|
| Depression (DRPST [1]): In recent one month, do you have the following symptoms persisted more than two weeks? | Item 1: Depressed mood most of the day, nearly every day. | 0 = no, 1 = yes |
| | Item 2: Fatigue or loss of energy nearly every day. | 0 = no, 1 = yes |
| | Item 3: Feeling of worthlessness or excessive or inappropriate guilt nearly every day | 0 = no, 1 = yes |
| Sleep (PSQI [2]): During the past month,…. | Item 1: Do you cannot get to sleep within 30 minutes? | 1 = not during the past month, 2 = less than once a week, 3 = once or twice a week, 4 = three or more times a week. |
| | Item 2: Do you wake up in the middle night or early morning, and you cannot sleep again? | 1 = not during the past month, 2 = less than once a week, 3 = once or twice a week, 4 = three or more times a week. |
| | Item 3: How would you rate your sleep quality overall? | 1 = very good, 2 = fairly good, 3 = fairly bad, 4 = very bad. |
| | Item 4: How much of a problem has it been for you to keep up enough enthusiasm to get things done? | 1 = not a problem at all, 2 = only a very slight problem, 3 = somewhat of a problem, 4 = a very big problem. |
| Physical pain (SF-12v2 [3]) | During the past one month, how much did pain interfere with your normal work (including both work outside the home and housework) | 1 = not at all, 2 = a little bit, 3 = moderately, 4 = quite a bit, 5 = extremely. |
| Social distance (SISQ [4]): How has COVID-19 impacted you? | Item 1: I avoid communication with or encountering strangers. | 1 = never (0%), 2 = Rarely (10%), 3 = sometimes (30%), 4 = often (60% or above) |
| | Item 2: I avoid close or personal contact with family members and/or people I am close to. | 1 = never (0%), 2 = Rarely (10%), 3 = sometimes (30%), 4 = often (60% or above) |
| | Item 3: I avoid going out, especially if I should require public transport | 1 = never (0%), 2 = Rarely (10%), 3 = sometimes (30%), 4 = often (60% or above) |
| | Item 4: I reduce eating out. | 1 = never (0%), 2 = Rarely (10%), 3 = sometimes (30%), 4 = often (60% or above) |

| | | |
|---|---|---|
| Social anxiety (SISQ [4]): How has COVID-19 impacted you? | Item 1: I worry about the pandemic affecting my work. | 1 = never (0%), 2 = Rarely (10%), 3 = sometimes (30%), 4 = often (60% or above) |
| | Item 2: I feel anxious or fearful due to the pandemic. | 1 = never (0%), 2 = Rarely (10%), 3 = sometimes (30%), 4 = often (60% or above) |
| Social information (SISQ [4]): How has COVID-19 impacted you? | Item 1: I constantly check for latest pandemic news updates via television, computer or phone | 1 = never (0%), 2 = Rarely (10%), 3 = sometimes (30%), 4 = often (60% or above) |
| | Item 2: I continuously seek out information regarding COVID-19. | 1 = never (0%), 2 = Rarely (10%), 3 = sometimes (30%), 4 = often (60% or above) |
| Social adaptation (SISQ [4]): How has COVID-19 impacted you? | Item 1: I am more cautious of residents from severely impacted areas | 1 = never (0%), 2 = Rarely (10%), 3 = sometimes (30%), 4 = often (60% or above) |
| | Item 2: I avoid or cancel traveling overseas (Answer "60%", if you have cancelled an overseas trip. Answer "10%" or "30%", if you are still considering your cancellation.) | 1 = never (0%), 2 = Rarely (10%), 3 = sometimes (30%), 4 = often (60% or above) |

[1]: DRPST= Disaster-Related Psychological Screening Test
[2]: PSQI= Pittsburgh Sleep Quality Index
[3]: SF-12v2= The 12-item Short Form Survey version 2
[4]: SISQ= Societal Influences Survey Questionnaires

**Supplementary Table S2 Distribution of marital status.**

| Marital status | n | % |
|---|---|---|
| Single | 64 | 53.8 |
| Married | 16 | 13.4 |
| Divorced | 33 | 27.7 |
| Widowed | 6 | 5.0 |
| Cohabited | 0 | 0 |

**Supplementary Table S3 Distribution of events for psychological trauma.**

| Psychological trauma | n | % |
|---|---|---|
| Biological disaster | 34 | 28.6 |
| Earthquake | 25 | 21.0 |
| Typhoon/ Flood/ Landslide | 20 | 16.8 |
| Kaohsiung Gas Explosion | 6 | 5.0 |
| Domestic violence in marriage | 26 | 21.8 |
| Victim of sexual assault | 10 | 8.4 |
| Childhood abuse | 16 | 13.4 |
| Unintentional accident | 8 | 6.7 |
| Military exercise/ war | 3 | 2.5 |
| Criminal events/ violence | 14 | 11.8 |
| Suicide/ self-harm | 25 | 21.0 |
| Others | 16 | 13.4 |

**Supplementary Table S4 Distribution of chronic disease (medical).**

| Chronic medical disease | n | % |
|---|---|---|
| Hypertension | 27 | 22.7 |
| Dyslipidemia | 18 | 15.1 |
| Diabetes mellitus | 17 | 14.3 |
| Coronary artery disease | 19 | 16.0 |
| Hepato-biliary disease | 6 | 5.0 |
| Gastric disease | 17 | 14.3 |
| Lung disease | 3 | 2.5 |
| Cancer | 1 | 0.8 |
| Others | 22 | 18.5 |

**Supplementary Table S5.** Predictors for level of depression verified with multivariate linear regression with 1000 bootstrapping samples.

| Predictors | β | 95% CI | p |
|---|---|---|---|
| Social anxiety | 0.09 | -0.02, 0.19 | 0.093[a] |
| Sex | | | |
| Male | Ref | - | - |
| Female | 0.45 | 0.06, 0.89 | **0.043** |
| Marital status | | | |
| Without partner | Ref | - | - |
| With partner | 0.67 | 0.08, 1.23 | **0.025** |
| Psychological trauma | | | |
| No | Ref | - | - |
| Yes | 0.58 | 0.07, 1.07 | **0.023** |
| Drinking (≥3 times per week) | | | |
| No | Ref | - | - |
| Yes | 0.70 | 0.03, 1.32 | **0.026** |

[a]: excluded from bootstrapping methods

**Supplementary Table S6.** Predictors for level of sleep disturbance verified with multivariate linear regression with 1000 bootstrapping samples.

| Predictors | β | 95% CI | p |
|---|---|---|---|
| Social anxiety | 0.33 | -0.07, 0.702 | 0.112 [a] |
| Social information | -0.02 | -0.43, 0.42 | 0.952 [a] |
| Social adaptation | 0.23 | -0.14, 0.59 | 0.234 [a] |
| Sex | | | |
| Male | Ref | | |
| Female | 1.27 | -0.01, 2.58 | 0.06 [a] |
| Marital status | | | |
| Without partner | Ref | | |
| With partner | 2.38 | 0.30, 4.58 | **0.038** |
| Drinking (≥3 times per week) | | | |
| No | Ref | | |
| Yes | 2.51 | 0.94, 3.94 | **0.002** |

[a]: excluded from bootstrapping methods

**Supplementary Table S7.** Predictors for level of physical pain verified with multivariate linear regression with 1000 bootstrapping samples.

| Predictors | β | 95% CI | p |
|---|---|---|---|
| Social anxiety | 0.21 | 0.05, 0.35 | **0.006** |
| Social information | 0.02 | -0.14, 0.18 | 0.768 [a] |
| Social adaptation | -0.04 | -0.17, 0.09 | 0.543 [a] |
| Chronic disease (medical) | | | |
| No | Ref | | |
| Yes | 0.38 | -0.05, 0.82 | 0.089 [a] |
| Psychological trauma | | | |
| No | Ref | | |
| Yes | 0.52 | 0.10, 0.94 | **0.019** |

[a]: excluded from bootstrapping methods
